# A Decade of Research on Coffee as an Anticarcinogenic Beverage

**DOI:** 10.1155/2021/4420479

**Published:** 2021-09-15

**Authors:** Ayelén D. Nigra, Anderson J. Teodoro, Germán A. Gil

**Affiliations:** ^1^Departamento de Química Biológica Ranwel Caputto, Facultad de Ciencias Químicas, Universidad Nacional de Córdoba-CIQUIBIC, CONICET, Córdoba, Argentina; ^2^Universidade Federal do Estado do Rio de Janeiro, Laboratory of Functional Foods, Rio de Janeiro, CEP 22290-240, Brazil

## Abstract

Coffee consumption has been investigated as a protective factor against cancer. Coffee is a complex beverage that contains more than 1000 described phytochemicals, which are responsible for its pleasant taste, aroma, and health-promoting properties. Many of these compounds have a potential therapeutic effect due to their antioxidant, anti-inflammatory, antifibrotic, and anticancer properties. The roasting process affects the phytochemical content, and undesirable compounds may be formed. In recent years, there have been contradictory publications regarding the effect of coffee drinking and cancer. Therefore, this study is aimed at evaluating the association of coffee consumption with the development of cancer. In PubMed, until July 2021, the terms “Coffee and cancer” resulted in about 2150 publications, and almost 50% of them have been published in the last 10 years. In general, studies published in recent years have shown negative associations between coffee consumption and the risk or development of different types of cancer, including breast, prostate, oral, oral and pharyngeal, melanoma, skin and skin nonmelanoma, kidney, gastric, colorectal, endometrial, liver, leukemic and hepatocellular carcinoma, brain, and thyroid cancer, among others. In contrast, only a few publications demonstrated a double association between coffee consumption and bladder, pancreatic, and lung cancer. In this review, we summarize the *in vitro* and *in vivo* studies that accumulate epidemiological evidence showing a consistent inverse association between coffee consumption and cancer.

## 1. Introduction

Drinking coffee has often been discouraged, due to its association with the description of negative effects, mainly attributed to caffeine. Consumers' beliefs about the effects of coffee are strongly influenced by this idea: only 25%, a relevant minority of consumers, think that drinking coffee could have beneficial effects on health [1]. A survey performed by the World Cancer Research Fund (WCRF) revealed that as many as 36% of health professionals thought drinking coffee increases the risk of cancer [2]. Despite the “bad reputation” that has been conferred on coffee for many years, it has been increasingly demonstrated that it offers numerous health benefits, ranging from reduced risk of several cancers to reduced risk of developing depression.

Coffee is widely consumed, ranking as one of the four most commonly consumed beverages in the world [3]. The generic name coffee covers about one hundred species of plants, cultivated in multiple countries with different types of weather conditions: Hawaii, Colombia, Jamaica, Ethiopia, Kenya, Brazil, Vietnam, and some African countries [4]. The coffee tree appertains to the Rubiaceae family, and there are more than 80 different species of the genus *Coffea L.* Globally, only two species are traded: *Coffea arabica*, accounting for 63% of the world production, and *Coffea canephora*, accounting for the rest of the world production [5]. As de Mejia et al. highlight, the species and the origin are only two of the factors that determine that we never drink two cups of coffee with the same chemical composition. Additionally, the final composition of coffee will depend on cultivation practices (traditional or organic), postharvest techniques (wet or dry), duration and conditions of storage, roasting degree (light, medium, or dark), kind of roasting process (standard or torrefaction), type of commercial coffee (roasted ground or instant), and grinding and brewing method (boiled, filtered, or espresso) [6].

The consumption of coffee may have a substantial effect on public health. In 2017, two large reviews based on meta-analyzes were published, which established that coffee consumption was more often associated with benefit than harm, a probable decrease in the risk of breast, colorectal, colon, endometrial, and prostate cancer; cardiovascular disease and mortality; Parkinson's disease; and type 2 diabetes [7, 8]. Then, it is no surprise that coffee motivates the interest of researchers and clinicians [9]. In PubMed, until April 2021, the search for the term “coffee” resulted in around 17,200 publications, including 1,430 reviews, 800 clinical trials, and 260 meta-analyses. However, the impact of coffee intake on chronic diseases, including cancer, has been a matter of debate in the last two decades. Accordingly, the search performed by combining both terms, “coffee and cancer”, resulted in about 2,150 publications, and almost 50% of them have been published in the last 10 years.

In this work, we present an update on the last decade of research related to coffee and its anticancer activity. For this purpose, a broad approach was used, as follows: (1) analysis of the main coffee compounds and their modifications by the roasting process, (2) review of the bioactivity of coffee extracts by *in vitro* assays, (3) analysis of the antioxidant activity of coffee and its components, and (4) data collection of large observational human studies.

### 1.1. Search Strategy

We searched on the PubMed database for basic and meta-analysis articles published from 2010 to June 2021. The search included the following relevant terms: “coffee” paired with “cancer,” “roasting,” “green,” “dark,” “bioactive,” “composition,” “*in vitro*,” “cell line,” “antioxidant,” “breast,” “leukemic,” “oral,” “oral and pharyngeal,” “gastric,” “non-melanoma skin,” “melanoma,” “endometrial,” “hepatocellular,” “prostate,” “esophageal,” “ovarian,” “colorectal,” “liver,” “brain,” “glioma,” “thyroid,” “bladder,” “pancreatic,” “laryngeal,” “lung,” “caffeine,” “polyphenol,” “Trigonelline,” “chlorogenic acid,” “cafestol,” “kahweol,” “caffeic acid,” “melanoidin,” “nicotinic acid,” “flavonoid,” and “N-methylpyridinium.”

No language restrictions were imposed. When available, priority was given to the conclusions of meta-analyses and systematic reviews. Within these studies, we selected those that reported statistically significant associations in their analyses, whether positive or negative.

## 2. The Main Bioactive Compounds of Coffee and Their Modifications by the Roasting Process

The traditional recommendation to reduce the consumption of coffee as a beverage or not to drink it at all because of a global risk profile has progressively given rise to a less negative view due to its better-known phytochemistry [10]. The knowledge that coffee and caffeine are not equivalent has increased the interest in discovering whether other components of coffee might contribute to the protective action in the human body [9]. Coffee is a complex beverage containing more than 1000 described phytochemicals responsible for its pleasant flavor, aroma, and health promoters [1, 6, 10]. Many of these compounds have therapeutic potential antioxidant, anti-inflammatory, antifibrotic, and anticancer effects.

As a consequence of roasting, profound changes occur in the chemical composition, which leads to the transformation of natural substances present in green beans into compounds derived from the Maillard reaction [4].  Table 1 shows the main compounds of green coffee, i.e., coffee beans before roasting, and the appearance of or increase in specific components in black coffee beans. This well-known roasting process involves the caramelization of carbohydrates and the pyrolysis of organic compounds. Here, we detail the main phytochemicals belonging to groups of sugars and sugar metabolites, protein and amino acids, fatty acids, chlorogenic acids (CGAs), organic acids, and other compounds.

“Bioactive compounds” are extra nutritional constituents that typically occur in small quantities in foods and have a positive effect on human health. These are also referred to as nutraceuticals, a term that reflects their existence in the human diet and their biological activity. They consist of a wide range of chemical compounds with different structures, physiological activities, and molecular mass between 200 and 1000 Da [11]. Green coffee beans have been shown to have high levels of bioactive compounds, but even after the roasting process and exposure to hot water, dark coffee maintains and develops numerous new phytochemicals that are beneficial to consumers' health [12].

As highlighted in Table 1, during the roasting process, the concentration of some nutraceutical compounds, such as 5-CQA, 3,4-diCQA, 3,5-diCQA, phenolic acid, trigonelline, polyphenolic, cafestol, and kahweol, decreases while others are formed, such as melanoidins, chlorogenic lactones, acid gallic, acid nicotinic, caffeic acid, flavonoids, and N-methylpyridinium (all of them marked in bold). This delicate balance between the formation and decomposition of bioactive compounds allows the biological effects of dark roasted coffee to be found in experimental models. Sometimes, these effects are higher in dark than lighter roasted coffee, despite the antioxidant content in dark coffee is lower [1]. In fact, Priftis et al. reported that in eight out of 13 coffee varieties, toasted coffee increased the free radical scavenging activity [13].

The five most abundant bioactive constituents in green coffee are *(I) caffeine*, which has been positioned by numerous studies as a protective agent for cell membranes against oxidative damage, with anticancer activity [4, 14, 15] and anti-inflammatory effects [16]; *(II) polyphenols*, which can cause a variety of important bioactivities with beneficial effects on human health [4]; *(III) trigonelline*, which has hypoglycemic, neuroprotective, antitumor (anti-invasive), antibacterial, and antiviral activities [3, 4, 17]; *(IV) chlorogenic acids* (CGAs), the most important class of polyphenols, which can be grouped into caffeoylquinic acids (CQA), feruloylquinic acids (FQA), and di-caffeoylquinic acids (diCQA), all of which are known to have powerful antioxidant, anticancer, anti-inflammatory, antibacterial, antipyretic, hepatoprotective, and neuroprotective effects and can help prevent retinal degeneration, obesity, and hypertension [4, 12, 18, 19]; and *(V) cafestol* and *kahweol*, main coffee diterpenes that have demonstrated anti-inflammatory, hepatoprotective, anticancer (tumor cell-inducing apoptosis and antiangiogenesis), antidiabetic, and antiosteoclastogenesis activities [20, 21].

In contrast, the main bioactive molecules in roasted coffee are *(I) caffeic acid*, with biological activities such as antitumor, antioxidation, anti-inflammatory, and immune regulation properties [4, 12, 17]; *(II) melanoidins*, whose nutritional antiradical, antioxidant, chelating, antimicrobial, antimutagenic, anticariogenic, antihypertensive, anti-inflammatory, and antiglycative properties have been described [22, 23]; (*III) nicotinic acid* (Niacin or Vitamin B3), which plays a role in DNA repair (interacting with PARP), has tumor suppressive effects, and inhibits cancer cell invasion (blocking epithelial-mesenchymal transition) [24, 25]; *(IV) flavonoids*, which exhibit a great diversity of biological activities, such as antioxidant, antiaging, anti-inflammatory, immunomodulation, cardioprotective, antibacterial, antiviral, antiparasitic, antihypertensive, antiulcerogenic, antidiabetic, and hepatoprotective properties, as well as prevention against cancer (including carcinogen inactivation, antiproliferation, cell-cycle arrest, induction of apoptosis, inhibition of angiogenesis, antioxidation, and reversal of multidrug resistance or a combination of these mechanisms) [26, 27]; and *(V) N-methylpyridinium*, identified as inducers of antioxidant response element pathway [28].

In summary, although *in vitro* and *in vivo* studies using individual components of coffee revealed multiple biological activities, the physiological properties of whole coffee will likely differ because coffee is a complex, nonstandardized beverage. Therefore, it is a variable mixture of hundreds of compounds, and its bioactivity may be influenced by possible matrix, synergistic, and/or antagonist effects. Additionally, only a small percentage of the ingested compounds may enter the circulatory system and reach the tissues, and very little of the absorbed material may retain the original structure present in the beverage [4]. For these reasons, it is worth pointing out that the prevention of various diseases derived from coffee consumption is usually the joint action of multiple components, and sometimes the synergistic effect of various types of compounds is much better than the activity of single compounds [29].

## 3. Bioactivity of Coffee Extracts, Evidence Provided by *In Vitro* Studies

Despite all the bioactivities described above for individual compounds isolated from coffee, in this work, we highlight the studies that consider whole -/coffee, because it has been shown that after a person drinks 2-3 cups of coffee, many components are metabolized (thus changing their original structures) or only reach transient and very low plasma concentrations [4, 12]. Additionally, it has not been possible to attribute the diverse and extensive bioactive capabilities of coffee to any pure compound.

In the last decade, numerous research groups have focused on the effect of coffee on tumors by conducting *in vitro* studies, to understand the bioactivities of this beverage in greater depth.  Table 2 presents many of these *in vitro* studies, which have addressed a great diversity of cancer types, coffee varieties, degrees of roasting [5, 19, 30, 31], and diverse effects including cell-cycle arrest, antiproliferative effects, and antiapoptotic and high antioxidant activities.

Remarkably, most of the investigations report a wide range of coffee effects on cancer cells. For example, studies on breast cancer cells focused on antiproliferative and antioxidant effects, cell-cycle arrest, induced apoptosis, regulation of gene expression, modulation of enzyme activity, and enhanced efficiency of cancer treatments [30, 32–34]. Studies on prostate cancer cells reported antiproliferative effects, cell-cycle arrest, apoptosis, and high antioxidant capacity [5, 19, 31, 32]. Research on kidney cancer cells found antiproliferative and antimigratory effects, EMT downregulation, and gene downregulation [35]. Furthermore, antiproliferative effects were reported in esophageal, urinary, bladder, lung, colon, oral, osteosarcoma, and glioblastoma cancer cells [12, 18, 30, 36].

Additionally, as Samoggia and Riedel and Montenegro et al. pointed out, intake of green coffee-based beverages has become popular in recent years due to the belief in their beneficial antioxidant properties [1, 31]. Some researchers have observed that green coffee treatments have antiproliferative activity, though not as powerful as that of roasted coffee treatments [12]. However, further cell experiments should be conducted to evaluate the deep molecular mechanisms and pharmacokinetics involved in the effects observed. The bioavailability and bioaccessibility of extracts should be investigated to determine the quantity of coffee required to achieve such effects, due to possible losses during digestion, absorption, and metabolization by the gut microbiota. Toxicity assays should be performed to ensure safety. In addition, *in vivo* and clinical tests would be required to recommend the consumption of different coffee types to help protect against cancer.

## 4. Antioxidant Properties of Coffee

As highlighted in the previous sections, among the main biological activities of the beverage and its components, the antioxidant activity was made evident in numerous investigations, both in chemical tests and different tumor cell cultures.

When, in aerobic life and our metabolism, the electron flow becomes uncoupled (transfer of unpaired single electrons), free radicals are continuously produced by the body's normal use of oxygen such as respiration. Oxygen-centered free radicals or ROS radicals are superoxide, hydroxyl, peroxyl, alkoxyl, hydroxyl radical, nitric oxide, and lipid hydroperoxides, while ROS nonradicals are singlet oxygen, hydrogen peroxide, and hypochlorous acid. Therefore, these intermediates, also called oxidants or prooxidants, can easily initiate the peroxidation of membrane lipids [37]. The imbalance towards high ROS concentration could result in oxidative damage to critical cellular biopolymers (proteins, lipids, and nucleic acids), especially if the free radicals are produced and accumulate unchecked for a prolonged period. The sustained oxidative damage of these biological macromolecules is linked to the development of chronic diseases like cancer.[38]. The reason why antioxidants are thought to be beneficial to our health is that ROS have been shown to be involved in many disease processes, including cancer [39].

In terms of food, an antioxidant has been defined as any substance that, when present at lower concentrations than those of an oxidizable substrate, significantly delays or inhibits the oxidation of the substrate. In recent years, there has been great interest in identifying alternative natural and safe sources of food antioxidants, especially those of plant origin. Nowadays, there is a growing interest in substances exhibiting antioxidant properties, which are supplied to human organisms as food components or as specific preventive pharmaceuticals [37]. The possibility of complementing the body's natural antioxidant defense system with exogenous antioxidants has continued to receive significant research attention as a result of their potential for wide applications [38].

In this sense, the antioxidant activity of coffee and of many of its components has been demonstrated [29, 40–43]. Additionally, recent studies have shown that coffee components can trigger tissue antioxidant gene expression and protect against gastrointestinal oxidative stress [44].

Recent studies conclude that coffee infusions significantly extend the chronological lifespan of the Saccharomyces cerevisiae yeast cells by protecting cells against reactive oxygen species, double DNA-strand break, and the decrease in metabolic activity [45]. However, the bibliography currently gives contradictory information as to whether slightly roasted or dark coffee generates greater antioxidant activity. Water extracts from green coffee are characterized by significant antioxidant properties and a high capacity to reduce transition metal ions because it contains many polyphenolic compounds that oxidize in the potential range tested [19, 46]. Jung et al. found that the cellular antioxidant activity of coffee extracts (in AML-12 and RAW 264.7 cells) has physiological antioxidant and anti-inflammatory activities, and that these effects are negatively correlated with roasting levels in the cell models [47]. Bobková et al. also support this theory, since they established that total antioxidant capacity reached the highest values in light roasted coffee, and the roasting process affected both the oxidative activity and the polyphenolic content [48]. Schouten et al. confirmed that acrylamide levels and antioxidant activity reached a maximum in the first coffee roasting degrees and then decreased as the heating process continued, both in Arabica and Robusta samples [49]. Wolska et al. showed that the method of brewing Arabica coffee and green coffee (simple infusion, espresso maker, French press, overflow espresso, or Turkish coffee) significantly affected the antioxidant potential of infusions [42]. In other research, antioxidant activity significantly rose with the degree of roasting, where strongly roasted coffee had higher activity than lightly roasted coffee [50].

As mentioned above, many coffee micronutrients are considered bioactive due to their high antioxidant capacity. Investigations have revealed that crude caffeine possesses hydrophilic antioxidant activity (145 𝜇mol Trolox equivalent (TE)/g) and lipophilic antioxidant activity (66 𝜇mol TE/g) [51], and its administration has led to the inhibition of the cyclooxygenase-2 enzyme [40]. The antioxidant activities of CGA exhibited protection against oxidative damage of macromolecules such as DNA, lipids, and proteins. When administered to mice under scopolamine-induced amnesia, CGA showed a neuroprotective function via the inhibition of acetylcholinesterase [40]. Coffee melanoidins, compounds formed during coffee roasting, have also been demonstrated as potential food ingredients due to their antioxidant properties. The antioxidant activity of these compounds, evaluated *in vitro*, was 725-750 *μ*mol Trolox/g, and it was tripled by the addition of sugar during coffee roasting, namely, torrefaction, known to increase the content of melanoidins [41]. Czachor et al. provide strong evidence that coffee flavonoids are responsible for scavenging free radicals and leading to longevity in yeast lacking Sod1, Sod2, and Rad52 proteins [45].

## 5. Have We Been Drinking a Medicine Daily for Hundreds of Years without Knowing It?

In recent years, the number of consistent epidemiological evidence of coffee and cancer has accumulated but due to similar methodological limitations, it is sometimes unsatisfactory. Therefore, interest has focused on meta-analyses or pooled analyses to bypass the shortage of individual studies [10].

The database of human meta-analysis studies is more abundant today than ever before. Among them, there is a great diversity and due to contradictions between them or the limited scope of their conclusions, they are often not considered, or their interpretation is difficult. For this reason, in this review, we endeavored to make an exhaustive summary of meta-analysis studies (78 out of 115 published in PubMed until April 2021), which found a significant association (positive or negative) between coffee consumption and different types of cancer development, treatment, or advancement.

Table 3 shows meta-analyses, cohort, or prospective studies published between 2010 and 2021. Importantly, the large number of patients involved in these studies provides evidence on the effect of years of coffee consumption on humans. We collected and analyzed more than 75 studies (as shown in Table 3), the vast majority of which (63) reported beneficial effects of coffee consumption, such as less development, metastasis, or mortality from cancer of different origins.

Most publications showed a negative association between coffee consumption and the risk or development of different types of cancer (Table 3, italic rows); these include breast, oral, oral, and pharyngeal, melanoma, skin and skin nonmelanoma, prostate, colorectal, endometrial, liver, leukemic and hepatocellular carcinoma, brain, and thyroid cancer among others (18 cancer types). Instead, a dual association was observed in bladder, gastric, pancreatic, and lung cancer, although only a few publications demonstrated this association. Surprisingly, we found that only 12 publications showed positive associations between cancer and coffee consumption (bold rows), as observed in a few types of cancer, including lung, bladder, pancreatic, laryngeal, and gastric cancer.

Although caffeine has been shown to have anticancer activity both *in vivo* and *in vitro*, many epidemiological studies have published evidence indicating that the risk of cancer decreased even in people who ingested decaffeinated coffee [43, 52–58]. In this sense, Hall et al. discovered that the amounts of many of the bioactive components, such as caffeic acid, chlorogenic acid, ferulic acid, pyrogallic acid, and trigonelline, did not change when decaffeinated coffee was compared to caffeinated coffee [59].

This update reveals a growing body of statistically significant evidence from epidemiological studies, suggesting that coffee drinking in most people (including different sexes, ethnicity, and ages) is beneficial and inversely associated with cancer risk [6, 60]. The fact that we found few publications on the positive association of coffee consumption and cancer does not imply that it is less important but rather suggests that more research should be carried out, as this would make it possible to formulate a clearer hypothesis.

Undoubtedly, moderate coffee consumption of up to 4 cups/day can be enjoyed as part of a healthy, balanced diet and an active lifestyle [9], since health claims associated with its consumption are broad enough to recommend it as a protective beverage. Probing for the detrimental effects of coffee should be focused on further drawing a conclusive approach for end-users to eliminate the ambiguities [29].

Higher doses of coffee have higher benefits in terms of risk reduction. However, further biological and epidemiological studies are required to determine the exact mechanism and analyze the specific subgroups [61]. In addition, it is important to recommend caution to avoid the concomitant use of coffee with drugs that have a significant interaction with coffee. There should be an adequate time interval between the intake of drugs and coffee based on the properties of the drugs. Pharmacists and physicians must be aware of the potential risks of drug-coffee interaction and counsel patients appropriately. Further *in vitro* and *in vivo* studies should be performed on frequently prescribed drugs to obtain robust evidence of the pharmacokinetic interaction with coffee [62].

## 6. Conclusions

In the last decade of research, the overall view on the impact of coffee on health has shifted from mostly detrimental outcomes towards a beneficial profile. In fact, the data on cancer disease are mostly balanced towards beneficial effects. The subsequent production of more clinical data with a higher number of cases, together with a better understanding of the components of coffee, has contributed to changing this perspective. It can be concluded according to the current knowledge that the labeling of coffee as a mostly unhealthy beverage lacks scientific support.

Throughout this review, we have detailed the main components of coffee, their variation in the roasting process, and the bioactivity of many of them. We provide a wealth of up-to-date information on the existing experimental evidence for the anticarcinogenic effects of this beverage and a comprehensive review of the important significant evidence for these effects on humans. We have summarized the accumulating epidemiological evidence pointing towards a consistently inverse association between coffee consumption and the risk of breast, oral, pharyngeal, melanoma, endometrial, hepatocellular, prostate, colorectal, liver, and brain cancers, among others.

The prospects for new bioactive compounds and the development of new drugs are focused on natural products. Considering the wide range and depth of evidence already collected, coffee, a beverage that has been widely included in the human diet for years, could be used not only for its well-known taste and stimulant, pleasurable effects, but also for social, pharmaceutical, and clinical purposes, as it provides health benefits and significant effects on cancer treatments.

More research is needed to find the right dosage and balance between the beneficial health effects of coffee (such as anticancer and antioxidant activities) and those traditionally considered negative. However, many of the “harmful” effects noted in antiquity can be attributed to excessive consumption or specific compounds (such as caffeine), which can be regulated or even eliminated from the beverage, thus minimizing the associated risks, without necessarily affecting the important beneficial effects described by the numerous research studies included in this review.

## Figures and Tables

**Table 1:** Compounds that increase or decrease during the coffee bean roasting process. The left column shows the most abundant compounds in green coffee that are lost during the roasting process, while the right column shows those that appear during the roasting process. The known bioactive compounds are marked in bold.

Coffee bean roasting process	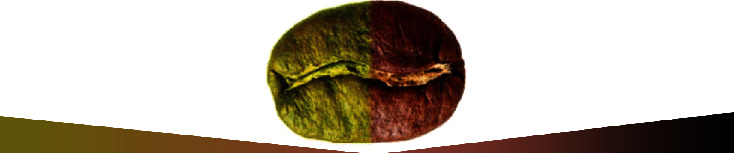	
Polysaccharide sugars and sugar metabolites	Sucrose [3, 19, 63–66], fructose [4, 19, 66], arabinos, galactose, glucose [4, 66], mannose, manitol, xylose, ribose [4]	Hydroxymethyl-furfural [3, 63, 64]
Proteins and amino acids	Asparagine [4, 19, 64], proline, glutamine, histidine, leucine, phenylalanine [19], alanine [4, 64]	Protein [66]
Lipids Fatty acids	Palmitic, stearic [4, 33], arachidic, linolenic, oleic, arachidic [4, 33]	(PUFA) n-6, MUFA+PUFA [33]
Polyphenols Chlorogenic acid (CGA)	1-FQA [66], 4-CoQA [66], 4-FQA [66, 67], 3-CQA, **5-CQA**, 4-CQA 4, [64, 66, 67], 5-FQA [66, 67], 4,5-diCQA [67], **3,4-diCQA**, **3,5-diCQA** [66, 67]	3-CoQA, 3-FQA, 5-CoQA [66], **chlorogenic lactones** [4]
Organic acids	**Citric**, GABA, malic [64], quinic [4, 64], **phenolic** [4, 14, 19]	**Gallic** [12, 63], **caffeic** [4, 12, 63], acetic [63], formic, lactic [63, 64], syllo-quinic [64], **nicotinic** (**Niacin** and **Vitamin B3)** [3, 4, 64]
Other compounds	**Caffeine** [3, 4, 19, 64, 66], **trigonelline** [3, 4, 64], **polyphenolic content** [13, 48], choline [3, 64], **cafestol**, **kahweol** [68], fluoride [42]	*γ*-Quinide [4, 64], **flavonoids** [69], **N-methylpyridinium** [3], alkyl esters, shikimoyl esters [4], **melanoidins** [3, 4, 13]

**Table 2:** *In vitro* studies in cell lines. Effects of different types of coffee on a broad spectrum of human cancer cell lines.

Author, date (year)	Cell lines	Cancer type	Coffee variety	Coffee type	IC_50_	Effects
Pounis, 2017 [32]	PC-3 DU145	Prostate	Uninformed	Roasted	Uninformed	Antiproliferative
Montenegro, 2021 [5, 31]	PC-3 DU145	Prostate	Arabica Brazil	Roasted Green	1-5 mg/mL	Cell-cycle arrest Antiproliferative, apoptosis, high antioxidant capacity
Palmioli, 2017 [70]	MDA-MB-231	Breast	Arabica (Brazil, Burundi and Colombia) Robusta (Uganda, Vietnam, and Tanzania)	Roasted Green	121 ± 10 ng/*μ*l 72 ± 7-315 ± 17 ng/*μ*l	Antiproliferative
Amigo-Benavent, 2017 [18]	OE-33 T24 A549	Esophageal Urinary bladder Lung	*Coffea arabica L.* (Colombia)	Green	01-1 mg/mL	Antiproliferative
Bauer, 2018 [19]	DU-145	Prostate	*Coffea canephora* var. Robusta (Brazil)	Dark, medium, light, green	Uninformed	Antiproliferative, induced apoptosis
Mojica, 2018 [12]	HT-29 SCC-25	Colon Oral	Columbia Supremo	Geen, cinnamon, city, full city, full city plus	Uninformed	Antiproliferative, antioxidant activity
Funakoshi-Tago 2020 [71]	MCF-7	Breast	Columbia Arabica Japan	Roasted coffee	2.5, 5 v/v%	Cell-cycle arrest, apoptosis, enhances tamoxifen proapoptotic activity.
Makino 2021 [35]	ACHN Caki-1	Kidney	Arabica or Robusta Coffee Japan	Roasted	Uninformed	Antiproliferative, antimigratory, apoptosis
Oleaga, 2012 [36]	HT29 MCF-7	Colon Breast	Instant caffeinated coffee		Uninformed	Cyclin D1, STAT5B, and ATF-2 downregulated
Nigra, 2021 [30]	MDAMB-231 MCF-7 HCT116 U2OS T98G	Breast Colon Osteosarcoma Glioblastoma	Coffea canephora var. Robusta Brazil	Roasted green	1 mg/mL	Antiproliferative, cell-cycle arrest, induced apoptosis, mitochondrial dysfunction

**Table 3:** Coffee consumption and cancer risk. 78 meta-analyses, cohort, or prospective studies, published between 2010 and 2021, which reported some type of significant association between coffee consumption and different cancer types, were analyzed. Italic rows: studies that report a negative association between coffee and cancer risk; bold rows: studies that report a positive association between coffee and cancer risk. Abbreviations: Pt: participants; Cn: control; Cn: cancer cases; c/d: cups/day.

First author, date	Type of cancer	Study group	Date of completion of included data	Observed effect on coffee consumption
Yu, 2011 [72]	Breast	40 prospective cohort studies (2,179,126 Pt and 34,177 Cc)	March 2010	Meta-analysis showed coffee drinking had an inverse association with cancer.
Li, 2013 [73]		16 cohort and 10 case-control studies (49,497 Cc)	July 2012	An inverse association was observed in ER^−^ negative subgroup.
Jiang, 2013 [74]		37 articles (966,263 Pt and 59,018 Cc)	December 2012	A strong and significant association with cancer risk was found for BRCA1 mutation carriers. The risk of breast cancer decreased by 2% for every 2 days.
Lowcock, 2013 [52]		1 cohort study (3,427 Cn and 3,062 Cc)	2002-2003	High coffee consumption, but not total caffeine, may be associated with reduced risk of ER^−^ and postmenopausal cancers.
Simonsson, 2013 [75]		1 preoperative study (634 Pt)	2002-2008	Tamoxifen-treated patients with ER^+^ tumors who consumed 2 or more c/d had significantly decreased risk for early events.
Rosendahl, 2015 [76]		1 cohort (1,090 Pt with invasive primary cancer)	2002-2012	A moderate (2–4 c/d) to high (≥5 c/d) coffee intake was associated with smaller invasive primary tumors and a lower proportion of ER^+^ tumors.
Lafranconi, 2018 [77]		21 prospective studies	March 2017	Coffee intake was associated with a 10% reduction in postmenopausal cancer risk.
Sánchez-Quesada, 2020 [78]		1 cohort study (10,812 Pt)	Uninformed	Among postmenopausal women, more than 1 c/d of coffee was associated with a lower incidence of cancer.

Yu, 2011 [72]	Leukemic	40 prospective cohort studies (2,179,126 Pt and 34,177 Cc)	March 2010	It confirmed that coffee consumption is associated with a reduced risk of cancer.

Zhang, 2015 [79]	Oral	12 studies (1,872,231 Pt and 4,037 Cc)	March 2015	Higher consumption might reduce the risk of cancer, especially in Europe.
Li, 2016 [80]		11 case-control and 4 cohort studies (2,832,706 Cn and 5,021 Cc)	2015	A protective benefit in oral cancer
He, 2020 [81]		14 case-control and 5 cohort studies (6456 Cc)	September 2018	High and intermediate versus low coffee intake was associated with a reduced risk of cancer. Coffee intake might have protective effects against cancer.
Farvid, 2021 [82]		2 preoperative studies (8900 Cc)	1980-2010 1991-2011	>3 c/d of coffee was associated with a 25% lower risk of cancer. Among cancer survivors, higher postdiagnostic coffee consumption was associated with better cancer and overall survival.

Turati, 2011 [83]	Oral and pharyngeal	1 cohort and 8 case-control studies (2,633 Cc)	October 2009	Coffee drinking is inversely related to oral pharyngeal cancer risk.
Miranda, 2017 [84]		13 case-control and 4 cohort studies	August 2016	An inverse association between high consumption and the risk of both cancer types
Hildebrand, 2013 [85]		A prospective US cohort study (967,564 Cn and 868 Cc)	1982-2008	Intake of >4 c/d was associated with a 49% lower risk of cancer. Caffeinated coffee intake was inversely associated with oral/pharyngeal cancer mortality.

Vaseghi, 2016 [86]	Nonmelanoma skin	6 independent studies (320,370 Pt and 104,770 Cc)	January 2016	Caffeinated coffee might have chemopreventive effects dose-dependent effects against basal cell carcinoma
Caini, 2017 [87]		13 articles (37,627 Cc)	February 2016	A moderate protective effect against basal cell cancer development

Wang, 2016 [88]	Cutaneous melanoma	23 studies (2,268,338 Pt)	August 2015	The risk of cancer decreased by 3% and 4% for 1 c/d increment of total coffee and caffeinated coffee consumption, respectively.

Liu, 2016 [89]	Melanoma	2 case-control (846 Cc and 843 Cn) and 5 cohort studies (844,246 Pt and 5,737 Cc)	November 2015	Caffeinated coffee might have chemopreventive effects against cancer.
Yew, 2016 [90]		9 studies (927,173 Pt and 3,787 Cc)	September 2015	Beneficial effects of regular coffee consumption on cancer.
Micek, 2018 [91]		7 studies (1,418,779 Pt and 9,211 Cc)	March 2017	An increase in consumption of one c/d was associated with a 3% reduction in cancer risk. Coffee intake may be inversely associated with the incidence of melanoma.

Je, 2012 [92]	Endometrial	10 case-control and 6 cohort studies (6,628 Cc)	October 2011	Increased intake is associated with a reduced risk of cancer.
Zhou, 2015 [53]		13 articles (1,534,039 Pt)	May 2015	Risk decreased by 5% for every 1 c/d intake, 7% for every 1 c/d of caffeinated coffee intake, 4% for every 1 c/d of decaffeinated coffee intake, and 4% for every 100 mg of caffeine intake/d
Lafranconi, 2017 [93]		12 studies	March 2017	Increasing consumption by 4 c/d was associated with a 20% reduction in risk and a 24% reduction in postmenopausal cancer risk.
Lukic, 2018 [94]		12 cohort and 8 case-control studies (11,663 Pt and 2,746 Cc)	August 2016	Protective effect

Bravi, 2013 [95]	Hepatocellular carcinoma	8 cohort and 8 control studies (3,153 Cc)	September 2012	The risk of cancer is reduced by 40% for any coffee consumption vs. no consumption regardless of the subjects' sex.
Bai, 2016 [96]		11 studies (340,749 Cn and 2,795 Cc)	August 2015	An inverse association between coffee consumption and cancer risk was observed, with quantitative evidence.
Bravi, 2017 [97]		12 studies (3,414 Cc)	Uninformed	The meta-analysis provides a precise quantification of the inverse relation between coffee consumption and the risk of cancer.
Kennedy, 2017 [54]		18 cohorts (2,272,642 Pt and 2,905 Cc) and 8 case-control studies, (4,652 Cn and 1,825 Cc)	Uninformed	An extra 2 cups of caffeinated and decaffeinated coffee were associated with reductions of 27 and 14% in the risk of cancer. Increased consumption is associated with a reduced risk of cancer, including preexisting liver disease.

Discacciati, 2014 [98]	Prostate	3 case-control and 5 cohort studies	July 2013	Inversely associated with the risk of fatal cancer
Lu, 2014 [99]		12 case-control (9,461 Cn and 7,909 Cc) and 9 cohort studies (455,123 Pt)	June 2013	High (highest ≥4 or 5 c/d) consumption may not only be associated with a reduced risk of cancer but also inversely associated with fatal and high-grade cancer.
Cao, 2014 [100]		10 cohort studies (206,096 and Pt8,973 Cc)	June 2013	Coffee consumption may decrease the risk of cancer.
Zhong, 2014 [101]		12 case-control and 12 cohort studies (42,179 Cc)	July 2013	An increase of 2 c/d was associated with a 7% decreased risk of cancer. A significant inverse relationship was also found for fatal cases and high-grade cancers.
Huang, 2014 [102]		13 cohort studies	August 2013	A significant reverse association was found between highest versus none/lowest consumption and risk of cancer.
Liu, 2015 [103]		13 cohort studies (539,577 Pt and 34,105 Cc)	Uninformed	Coffee consumption may be associated with a reduced risk of cancer, and it also has an inverse association with nonadvanced cancer.
Xia, 2017 [104]		14 case-control and 14 cohort studies (42,399 Pt)	July 2016	An effect on reducing the localized cancer risk
Pounis, 2017 [32]		1 cohort study (6,989 Pt and 100 Cc)	March 2005-April 2010	Reduction of 53% lower cancer risk by Italian-style coffee consumption
Chen, 2021 [105]		16 cohort studies (1.081.586 Pt and 57,732 Cc)	September 2020	Higher coffee consumption was significantly associated with a lower risk of cancer.

Zheng, 2013 [106]	Esophageal	24 case-control and cohort studies (7,376 Cc)	October 2011	Borderline significantly inverse association of highest versus non/lowest consumption against risk (protective effects)
Zhang, 2018 [107]		11 studies (457,010 Pt and 2,628 Cc)	January 2017	An inverse association between coffee consumption and incidence of cancer was found in East Asian participants.

Shafiei, 2019 [55]	Ovarian	22 case–control and 20 studies (40,140 Pt)	April 2018	Inverse association between decaffeinated coffee consumption and risk of cancer

Galeone, 2010 [108]	Colorectal	24 studies (14,846 Cc)	May 2010	A moderate favorable effect on cancer risk
Li, 2013 [109]		25 case-control (15,522 Cc) and 16 cohort studies (10 443 Cc)	May 2011	Coffee consumption can significantly decrease the risks of colorectal and colon cancer, especially in Europe and for females.
Tian, 2013 [110]		21 studies case-control and 12 cohort studies	Uninformed	A significant association was found between consumption and decreased risk of colorectal and colon cancer among subjects consuming ≥4 c/d.
Gan, 2017 [60]		19 cohort studies (2,046,575 Pt and 22,629 Cc)	August 2015	Coffee consumption was significantly associated with a decreased risk of cancer at ≥5 c/d.
Nakagawa-Senda, 2017 [111]		2 case-control studies (13,480 Cn and 2,696 Cc)	1988 – 2000 2001-2005	The study found a significant inverse linear trend between consumption and distal colon cancer and a tendency toward a lower risk of rectal cancer.
Micek, 2019 [43]		14 prospective studies (1,381,085 Pt and 28,404 Cc)	August 2018	Restriction to decaffeinated coffee revealed a 15% lower risk of cancer for the highest category consumption. Coffee consumption was related with a decreased risk of cancer in a subgroup of never-smokers and in Asian countries.
Sartini, 2019 [56]		26 prospective studies	Uninformed	Regarding colorectal cancer, a protective effect emerged in US subjects. Concerning colon cancer, a significant protective effect was noted only in European men and only in Asian women. Decaffeinated coffee exhibited a protective effect against colorectal cancer in men and women combined.
Mackintosh, 2020 [57]		1 prospective observational cohort study (1171 Pt)	2005-2018	Increased consumption of coffee was associated with decreased risk of cancer progression. Significant associations were noted for both caffeinated and decaffeinated coffee.
Um, 2020 [58]		1 prospective cohort study (107,061 Pt and 1,829 Cc)	1999-2015	A higher intake of decaffeinated coffee was associated with a lower risk of colorectal, colon, and rectal cancer.

Sang, 2013 [112]	Liver	9 case-control and 7 cohort studies	May 2012	An inverse association was observed between coffee consumption and cancer.
Yu, 2016 [113]		20 cohort studies from 10 publications	Jan 2016	A significant linear dose-response relationship was found between consumption and cancer risk.
Godos, 2017 [114]		13 studies	March 2017	An inverse correlation was noted between consumption and cancer. Increasing consumption by 1 c/d was associated with 15% reduction in cancer risk.
Tamura, 2019 [115]		6 cohort studies from 5 publications	Uninformed	Consumption among Japanese people has a significant role in preventing cancer.
Tanaka, 2019 [116]		4 cohort and 4 case-control studies	September 2018	Coffee drinking decreases the risk of primary cancer among the Japanese population.
Bhurwal, 2020 [117]		20 prospective studies	June 2019	Higher doses of coffee consumption were associated with a significant decrease in the risk of developing cancer.

Song, 2019 [118]	Brain	11 articles	November 2018	A statistically significant protective effect of consumption and cancer risk was reported.

Creed, 2020 [119]	Glioma	1 prospective study (379,259 Pt and 487 Cc)	2006-2010	A suggestive inverse association was observed with greater consumption of coffee.
Pranata, 2021 [61]		12 studies (1,960,731 Pt and 2,987 Cc).	October 2020	Dose-response meta-analysis showed that every 1 c/d of coffee decreases the risk of glioma by 3%.

Shao, 2019 [120]	Thyroid	10 studies (379,825 Pt and 1,254 Cc)	February 2019	Inversely associated with cancer occurrence in a linear dose-response manner. The occurrence of cancer was reduced by 5% with each 1 c/d increment of coffee consumption.

Sugiyama, 2017 [121]	Bladder	2 cohort studies (73,346 Pt and 274 Cc)	Uninformed	A significant inverse association was observed between coffee consumption and the risk of cancer.

Wu, 2015 [122]		34 case-control and 6 cohort studies	Uninformed	An increased risk between coffee consumption and cancer was found.
Yu, 2020 [123]		12 cohort studies (2601 Cc and 501,604 Pt)	Uninformed	Positive associations are suggested between coffee consumption and cancer among male smokers but not among never-smokers and females.

Dong, 2011 [124]	Pancreatic	14 studies (669,584 Pt and 1,496 Cc)	August 2010	An inverse relationship was found between coffee drinking and the risk of cancer.
Ran, 2016 [125]		20 cohort studies	June 2015	High coffee consumption is associated with reduced risk.

Nie, 2016 [126]		20 articles	November 2015	Every 1-cup increase was associated with a 1% increase in risk. Coffee consumption may weakly increase the risk of cancer.
Li, 2019 [127]		13 cohort studies (959,992 Pt and 3,831 Cc)	February 2018	Coffee consumption is related to increased risk of cancer in a dose-response manner.

Xie, 2016 [128]	Gastric	9 cohort and 13 case-control studies (1,019,693 Cn and 7,631 Cc)	July 2014	An increase in consumption was associated with a decreased risk of cancer.

Shen, 2015 [129]		8 studies (311,564 Pt and 1,429 Cc)	October 2013	Coffee consumption is associated with the development of cancer. More coffee drinking could result in an increased risk of cancer.
Zeng, 2015 [130]		9 studies, 15 independent prospective cohorts (1,289,314 Pt and 2,019 Cc)	February 2015	High coffee consumption (>6.5 c/d) might increase the risk of cancer in the US population.
Deng, 2016 [131]		13 cohort studies (1,324,559 Pt and 3,484 Cc)	September 2014	High coffee consumption is a risk factor for cancer.

Chen, 2014 [132]	Laryngeal	10 studies (503,234 Cn and 2,803 Cc)	October 2013	Coffee consumption would increase cancer risk.

Kudwongsa, 2020 [133]	Lung	1 prospective cohort study (12,668 Pt and 138 Cc)	1990-2016	Coffee consumption was associated with a reduced risk of cancer. Consumption may be a protective factor for cancer among this cohort.

Tang, 2010 [134]		5 prospective and 8 case-control studies (104,911 Pt and 5347 Cc)	January 2009	Highest consumption was significantly associated with an increased risk of cancer.
Wang, 2012 [135]		9 publications (3,008 Cc)	2005	A significantly positive association was found between coffee consumption and the risk of cancer.
Xie, 2016 [136]		5 cohort and 12 case-control studies (102,516 Cn and 12,276 Cc)	March 2015	Cancer risk is significantly increased by 47% in the population with the highest category intake of coffee compared with that with the lowest category intake.
Zhu, 2020 [137]		17 prospective cohort studies (1.1 million Pt and 20,280 Cc)	Uninformed	Higher consumption of coffee is associated with increased cancer risk.

## Data Availability

The data used to support the findings of this study are available from the corresponding authors upon request.
